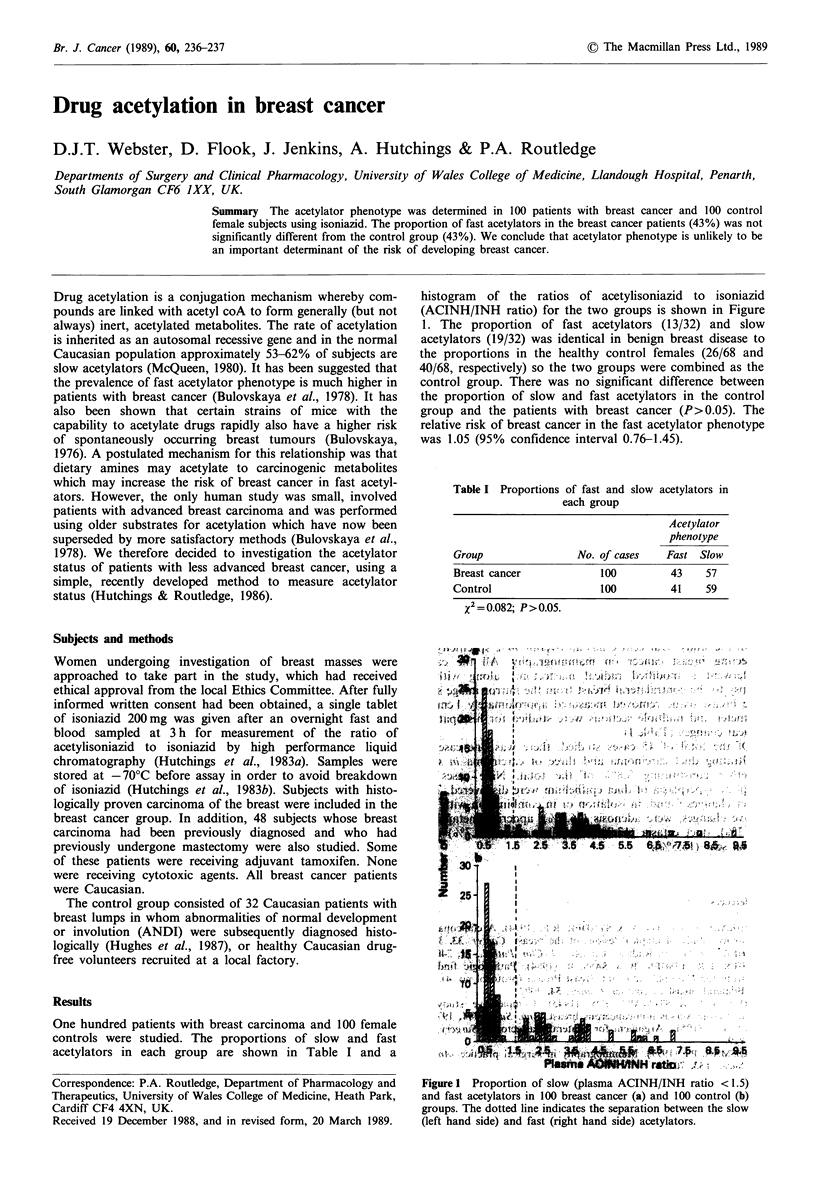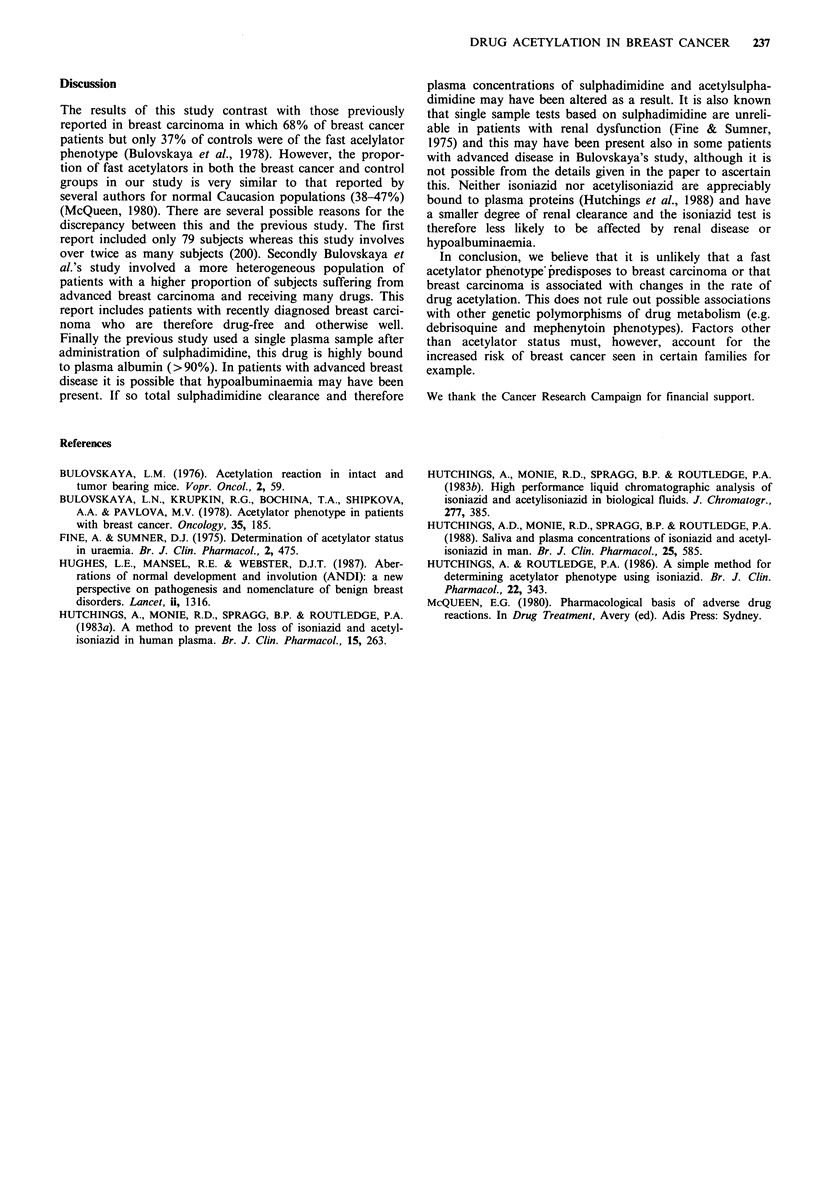# Drug acetylation in breast cancer.

**DOI:** 10.1038/bjc.1989.260

**Published:** 1989-08

**Authors:** D. J. Webster, D. Flook, J. Jenkins, A. Hutchings, P. A. Routledge

**Affiliations:** Department of Surgery, University of Wales College of Medicine, Llandough Hospital, Penarth, South Glamorgan, UK.

## Abstract

**Images:**


*Br. J. Cancer* (1989), **60**, 236–237 © The Macmillan Press Ltd., 1989

### Drug acetylation in breast cancer

D.J.T. Webster, D. Flook, J. Jenkins, A. Hutchings & P.A. Routledge

*Departments of Surgery and Clinical Pharmacology, University of Wales College of Medicine, Llandough Hospital, Penarth, South Glamorgan CF6 1XX, UK.*

**Summary** The acetylator phenotype was determined in 100 patients with breast cancer and 100 control female subjects using isoniazid. The proportion of fast acetylators in the breast cancer patients (43%) was not significantly different from the control group (43%). We conclude that acetylator phenotype is unlikely to be an important determinant of the risk of developing breast cancer.

Drug acetylation is a conjugation mechanism whereby compounds are linked with acetyl coA to form generally (but not always) inert, acetylated metabolites. The rate of acetylation is inherited as an autosomal recessive gene and in the normal Caucasian population approximately 53–62% of subjects are slow acetylators (McQueen, 1980). It has been suggested that the prevalence of fast acetylator phenotype is much higher in patients with breast cancer (Bulovskaya *et al.*, 1978). It has also been shown that certain strains of mice with the capability to acetylate drugs rapidly also have a higher risk of spontaneously occurring breast tumours (Bulovskaya, 1976). A postulated mechanism for this relationship was that dietary amines may acetylate to carcinogenic metabolites which may increase the risk of breast cancer in fast acetylators. However, the only human study was small, involved patients with advanced breast carcinoma and was performed using older substrates for acetylation which have now been superseded by more satisfactory methods (Bulovskaya *et al.*, 1978). We therefore decided to investigation the acetylator status of patients with less advanced breast cancer, using a simple, recently developed method to measure acetylator status (Hutchings & Routledge, 1986).

### Subjects and methods

Women undergoing investigation of breast masses were approached to take part in the study, which had received ethical approval from the local Ethics Committee. After fully informed written consent had been obtained, a single tablet of isoniazid 200 mg was given after an overnight fast and blood sampled at 3 h for measurement of the ratio of acetylisoniazid to isoniazid by high performance liquid chromatography (Hutchings *et al.*, 1983*a*). Samples were stored at −70°C before assay in order to avoid breakdown of isoniazid (Hutchings *et al.*, 1983*b*). Subjects with histologically proven carcinoma of the breast were included in the breast cancer group. In addition, 48 subjects whose breast carcinoma had been previously diagnosed and who had previously undergone mastectomy were also studied. Some of these patients were receiving adjuvant tamoxifen. None were receiving cytotoxic agents. All breast cancer patients were Caucasian.

The control group consisted of 32 Caucasian patients with breast lumps in whom abnormalities of normal development or involution (ANDI) were subsequently diagnosed histologically (Hughes *et al.*, 1987), or healthy Caucasian drug-free volunteers recruited at a local factory.

### Results

One hundred patients with breast carcinoma and 100 female controls were studied. The proportions of slow and fast acetylators in each group are shown in Table I and a histogram of the ratios of acetylisoniazid to isoniazid (ACINH/INH ratio) for the two groups is shown in Figure 1. The proportion of fast acetylators (13/32) and slow acetylators (19/32) was identical in benign breast disease to the proportions in the healthy control females (26/68 and 40/68, respectively) so the two groups were combined as the control group. There was no significant difference between the proportion of slow and fast acetylators in the control group and the patients with breast cancer ($P > 0.05$). The relative risk of breast cancer in the fast acetylator phenotype was 1.05 (95% confidence interval 0.76–1.45).

**Table I** Proportions of fast and slow acetylators in each group

| *Group* | *No. of cases* | *Acetylator phenotype* Fast | *Acetylator phenotype* Slow |
|---|---|---|---|
| Breast cancer | 100 | 43 | 57 |
| Control | 100 | 41 | 59 |

$\chi^2 = 0.082$; $P > 0.05$.

**Figure 1** Proportion of slow (plasma ACINH/INH ratio <1.5) and fast acetylators in 100 breast cancer (**a**) and 100 control (**b**) groups. The dotted line indicates the separation between the slow (left hand side) and fast (right hand side) acetylators.

Correspondence: P.A. Routledge, Department of Pharmacology and Therapeutics, University of Wales College of Medicine, Heath Park, Cardiff CF4 4XN, UK.
Received 19 December 1988, and in revised form, 20 March 1989.

### Discussion

The results of this study contrast with those previously reported in breast carcinoma in which 68% of breast cancer patients but only 37% of controls were of the fast acelylator phenotype (Bulovskaya *et al.*, 1978). However, the proportion of fast acetylators in both the breast cancer and control groups in our study is very similar to that reported by several authors for normal Caucasion populations (38–47%) (McQueen, 1980). There are several possible reasons for the discrepancy between this and the previous study. The first report included only 79 subjects whereas this study involves over twice as many subjects (200). Secondly Bulovskaya *et al.*'s study involved a more heterogeneous population of patients with a higher proportion of subjects suffering from advanced breast carcinoma and receiving many drugs. This report includes patients with recently diagnosed breast carcinoma who are therefore drug-free and otherwise well. Finally the previous study used a single plasma sample after administration of sulphadimidine, this drug is highly bound to plasma albumin (>90%). In patients with advanced breast disease it is possible that hypoalbuminaemia may have been present. If so total sulphadimidine clearance and therefore plasma concentrations of sulphadimidine and acetylsulphadimidine may have been altered as a result. It is also known that single sample tests based on sulphadimidine are unreliable in patients with renal dysfunction (Fine & Sumner, 1975) and this may have been present also in some patients with advanced disease in Bulovskaya's study, although it is not possible from the details given in the paper to ascertain this. Neither isoniazid nor acetylisoniazid are appreciably bound to plasma proteins (Hutchings *et al.*, 1988) and have a smaller degree of renal clearance and the isoniazid test is therefore less likely to be affected by renal disease or hypoalbuminaemia.

In conclusion, we believe that it is unlikely that a fast acetylator phenotype predisposes to breast carcinoma or that breast carcinoma is associated with changes in the rate of drug acetylation. This does not rule out possible associations with other genetic polymorphisms of drug metabolism (e.g. debrisoquine and mephenytoin phenotypes). Factors other than acetylator status must, however, account for the increased risk of breast cancer seen in certain families for example.

We thank the Cancer Research Campaign for financial support.

### References

BULOVSKAYA, L.M. (1976). Acetylation reaction in intact and tumor bearing mice. *Vopr. Oncol.*, **2**, 59.

BULOVSKAYA, L.N., KRUPKIN, R.G., BOCHINA, T.A., SHIPKOVA, A.A. & PAVLOVA, M.V. (1978). Acetylator phenotype in patients with breast cancer. *Oncology*, **35**, 185.

FINE, A. & SUMNER, D.J. (1975). Determination of acetylator status in uraemia. *Br. J. Clin. Pharmacol.*, **2**, 475.

HUGHES, L.E., MANSEL, R.E. & WEBSTER, D.J.T. (1987). Aberrations of normal development and involution (ANDI): a new perspective on pathogenesis and nomenclature of benign breast disorders. *Lancet*, **ii**, 1316.

HUTCHINGS, A., MONIE, R.D., SPRAGG, B.P. & ROUTLEDGE, P.A. (1983*a*). A method to prevent the loss of isoniazid and acetylisoniazid in human plasma. *Br. J. Clin. Pharmacol.*, **15**, 263.

HUTCHINGS, A., MONIE, R.D., SPRAGG, B.P. & ROUTLEDGE, P.A. (1983*b*). High performance liquid chromatographic analysis of isoniazid and acetylisoniazid in biological fluids. *J. Chromatogr.*, **277**, 385.

HUTCHINGS, A.D., MONIE, R.D., SPRAGG, B.P. & ROUTLEDGE, P.A. (1988). Saliva and plasma concentrations of isoniazid and acetylisoniazid in man. *Br. J. Clin. Pharmacol.*, **25**, 585.

HUTCHINGS, A. & ROUTLEDGE, P.A. (1986). A simple method for determining acetylator phenotype using isoniazid. *Br. J. Clin. Pharmacol.*, **22**, 343.

McQUEEN, E.G. (1980). Pharmacological basis of adverse drug reactions. In *Drug Treatment*, Avery (ed). Adis Press: Sydney.